# Case Report: Early detection of lung carcinoid in an asymptomatic individual by blood-test initiated PET-CT imaging

**DOI:** 10.3389/fonc.2023.1177237

**Published:** 2023-06-06

**Authors:** Simon Burg, Ralf Smeets, Martin Gosau, Katja Failing, Audrey Laure Céline Grust

**Affiliations:** ^1^ Department of Oral and Maxillofacial Surgery, University Medical Center Hamburg-Eppendorf (UKE), Hamburg, Germany; ^2^ Department of Oral and Maxillofacial Surgery, Division of “Regenerative Orofacial Medicine”, University Medical Center Hamburg-Eppendorf (UKE), Hamburg, Germany; ^3^ Precura Center, Darmstadt, Germany

**Keywords:** early cancer screening, Apo10/DNaseX, TKTL1, immunological biopsy, blood test, FDG-PET/CT

## Abstract

We present the case of a 53-year-old woman who was diagnosed with early-stage lung cancer by targeted cancer screening consisting of an immunological biopsy-based blood test followed by radiological imaging. The PanTum Detect blood test detects the biomarkers Apo10/DNaseX and Transketolase-like 1 (TKTL1) in circulating macrophage-like cells from peripheral blood samples to identify asymptomatic individuals with a high risk for malignancy. The elevated blood test values initiated an ^18^F-FDG PET/CT visualization for further clarification. In this case, imaging indicated a lung carcinoma in the right upper lobe. A biopsy confirmed the presence of a lung carcinoma, which was removed surgically. Histologic examination revealed a typical I A2 carcinoid, which was completely removed, making further therapy obsolete.

## 1 Introduction

Lung cancer is still one of the most common causes of cancer morbidity and mortality (1). It is generally well known that the success of a cancer therapy depends very decisively on the time of the diagnosis and on tumor characteristics such as invasiveness and therapy resistance. When detected at a localized stage, most entities can be cured by surgery alone and without further systemic therapy (2). Therefore, it is of great importance to establish diagnostic methods enabling early detection. Controlled trials with high-risk smokers and ex-smokers have shown that low-dose computed tomography (LDCT) can significantly reduce lung cancer mortality (3). Imaging modalities such as positron emission tomography using the glucose analogue 2-[18F]fluoro-2-deoxy-D-glucose (FDG-PET) in combination with computed tomography (FDG-PET/CT) have proven to be powerful tools for the localization and assessment of various tumor types (4–8). However, due to the cost and risk of radiation exposure, whole-body FDG-PET/CT does not yet appear suitable for general cancer screening in asymptomatic populations (4, 9). In contrast, a blood test that detects a variety of tumor types at early, asymptomatic stages can be used to pre-select subjects with a high likelihood of (pre)cancerous lesions on subsequent imaging (10). The PanTum Detect blood test is based on the unique technique of epitope detection in monocytes (EDIM) utilizing the fact that activated monocytes/macrophages (CD14+/CD16+), belonging to the group of circulating cancer-associated macrophage-like cells (CAMLs), phagocytose tumor cells and contain tumor proteins intracellularly (11–15). To prove internalization of tumor particles by CD14+/CD16+ monocytes, co-culture experiments were performed (16). These can be detected by flow cytometry from peripheral blood samples using specific antibodies. The PanTum Detect blood test screens for the biomarkers Apo10 and TKTL1, which identify basic biophysical mechanisms that are considerably altered in various forms of tumors (13, 17–19).

## 2 Case description

### 2.1 Patient information

The 53-year-old woman, 169 cm tall, weighing 77 kg (BMI 26.96) participated in the prospective non-interventional study “Pre-PanTum”, which was conducted between November 2020 and April 2021 at the Precura Center, Darmstadt, Germany (Ethics Committee of the Medical Association of Hessen, Frankfurt, Germany, approval number: 2020-1981-evBO). Anamnesis revealed a hot thyroid nodule, incipient osteoarthritis, cysts in the breast, neurodermatitis, arthritis of the left shoulder, hay fever, and various unspecified allergies. Since 01.07.2019, the subject has been medicated with 25 µg Levothyroxine (Thyronajod Henning, Sanofi-Aventis, 65926 Frankfurt/Main, Germany) per day. No other comorbidities, complaints, as well as current cancer diagnoses, or indications and symptoms suspecting a cancer disease were reported. She stated to have smoked about one cigarette per day for 7 years but being an ex-smoker for 24 years. Family history showed that her mother was diagnosed with ovarian cancer at age 75 and her uncle with colorectal cancer at age 67.

### 2.2 Clinical findings

The PanTum Detect blood test resulted in an elevated combined PanTum score of 270 (Apo10 score: 144, TKTL1 score: 126). All other tumor markers tested (CEA, CA 19-9, CA 125, CA 15-3, AFP, beta HCG, CRP) were not elevated. Further PET/CT imaging analysis was recommended for clarification.

### 2.3 Timeline

**Figure 1:**
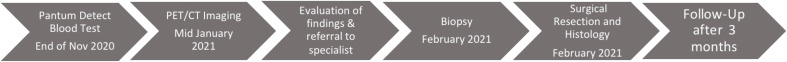
Timeline showing the diagnostic and therapeutic schedule from November 2020 until May 2021.

**Figure 1**. Timeline to demonstrate the diagnostic and therapeutic workflow from Pantum Detect blood test until required surgical resection and Follow-Up. The time from blood test sample receipt to complete surgical resection was 12 weeks.

### 2.4 Diagnostic assessments

#### 2.4.1 Blood collection and blood test analysis

At least 2 ml of EDTA whole blood was collected per subject after a minimum of 60 minutes of fasting using a Sarstedt Monovette 2.7 ml EDTA. Shipping to the laboratory was undertaken by a transport service provider specialized in shipping blood samples, and the samples were stored at room temperature (15-25°C). Staining was performed with antibodies CD14 (OFC-14D), CD16 (Hi-16a), Apo10 (clone JFC 19X63) and TKTL1 (clone JF- C12T10), and flow cytometry analysis took place within 36 hours after blood collection at PreMed Labor GmbH, Pfungstadt, Germany with a BD FACSCantoTM II Flow Cytometry (Canto) operating software BD FACSDivaTM software version 8.0.3 and version 9.0.1. Test results were considered positive if the sum of the two individual scores for Apo10 and TKTL1 reached or exceeded the threshold of 260 and, in addition, the Apo10 individual score was ≥ 140 (hereafter total score). In case of elevated values, further imaging was recommended for clarification.

#### 2.4.2 Imaging

For the assessment of the FDG-PET/CT, she was instructed to fast for a minimum of 6 hours and to avoid strenuous exercise 48 hours prior to the examination. She was injected with 97 MBq ^18^F-FDG and imaged on a Siemens Biograph Vision 600 PET/CT (Siemens Healthineers AG, 91052 Erlangen, Germany) in CT mode (153 mGy.cm). Following an uptake-phase (60 to 90 minutes) a low-dose CT was performed from the base of the skull to the thighs without iodinated contrast. Further details of the FDG-PET/CT imaging conditions can be found elsewhere (10).

**Figure 2:**
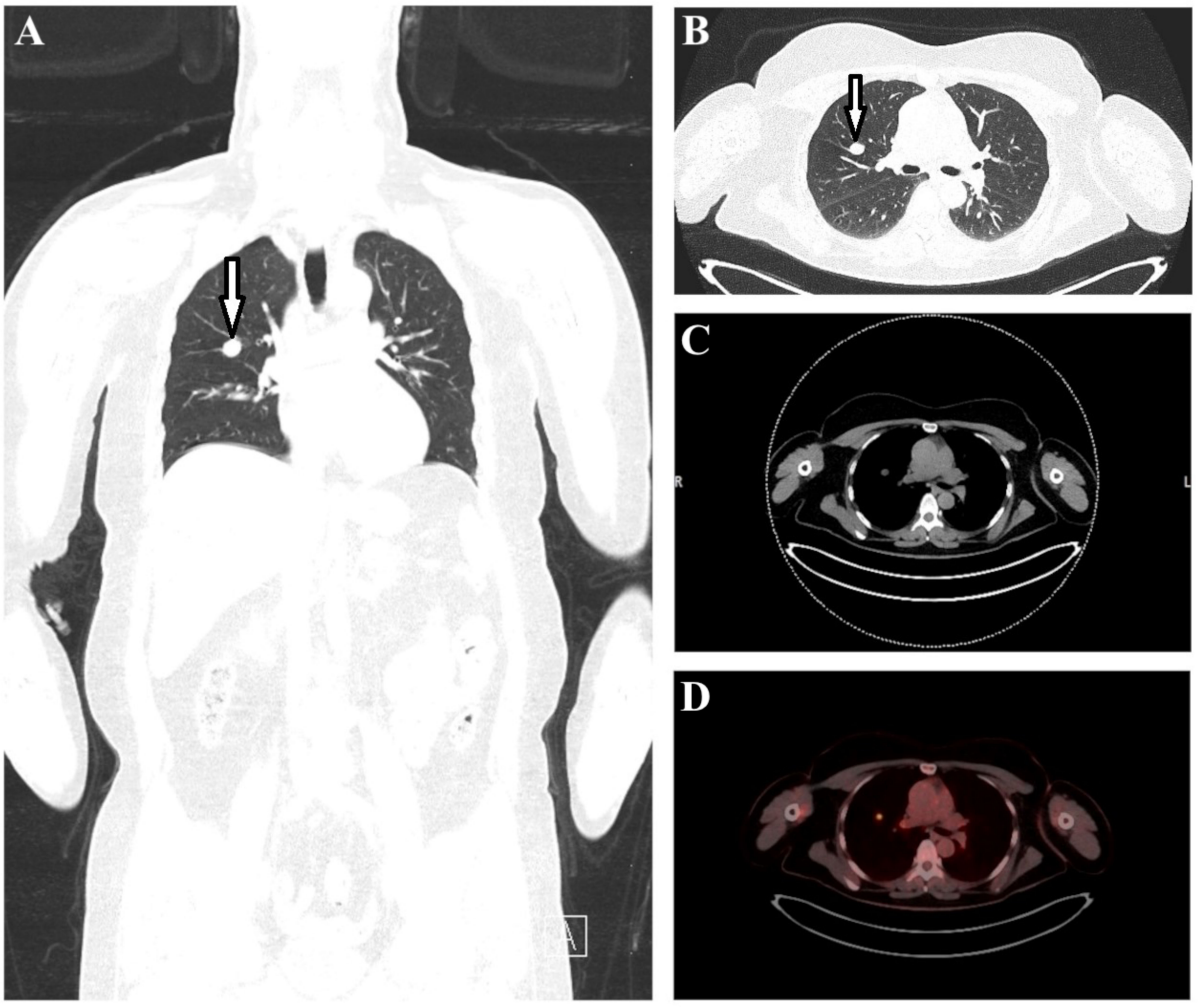
The multiplanar reformate image **(A)** and lung CT **(B)** revealed a 16 mm x 12 mm mass in the right upper lobe (marked with arrows). Axial CT **(C)** and transaxial fused PET-CT image **(D)** showed an increased focal ^18^F-FDG uptake (SUVmax 6.3) in the right upper lobe parahilar.

The multiplanar reformate image (MPR) and lung CT revealed a 16 mm x 12 mm mass in the right upper lobe (**Figures 2A, B**, marked by arrows). In accordance with this, axial CT soft tissue window (**Figure 2C**) and transaxial fused PET-CT image (**Figure 2D**) showed an increased focal ^18^F-FDG uptake (SUVmax 6.3) in the right upper lobe adjacent to the hilus. In addition, a bilateral diffuse enhancement with mild focal emphasis cranially of the hilus (left SUVmax 5.8 > right SUVmax 4.8) was detected. The radiological findings revealed an urgent suspicion of lung carcinoma in the right upper lobe parahilar without pathologic lymph nodes. For bioptic/histological clarification, the patient was then referred for presentation to a pneumological/thoracic surgery center.

### 2.5 Therapeutic intervention

Biopsy confirmed malignant lung carcinoma. The surgical resection and subsequent histologic examination revealed a typical stage I A2 carcinoid without nodes or metastases (I A2 ED 01/21 pT1b (1.2 cm), pN0 (0/38), L0, V0, Pn0, R0 cM0) of the right upper lobe. As the tumor was completely removed, no further therapy was required.

### 2.6 Follow-up

At a follow-up three months after surgery, the subject reported about an occasional paresthesia in the thoracotomy area. The surgical scar appeared irritation-free and healed well. The thoracic CT showed postoperative changes, with no evidence of tumor recurrence. No adverse or unanticipated events were recorded.

## 3 Discussion

The field of early cancer detection is becoming increasingly important, which is also reflected in the growing number of publications in this area in recent years.

In a prospective, interventional study, called DETECT-A the feasibility and safety of multicancer blood testing coupled with PET/CT was evaluated in 10,006 asymptomatic women (6). On the one hand, screening of cell-free DNA (cfDNA) and specific protein biomarkers can provide first hints on the tumor entity, on the other hand, the concentration of cfDNA in the blood must be sufficiently high for this (20). The EDIM technology used for the PanTum Detect blood test however uses the body’s own immune system to detect disease-specific epitopes inside macrophages and this represents a very sensitive and specific biological mechanisms, which can achieve a very high diagnostic performance.

A study meanwhile conducted with more than 5,000 participants indicates that the upstream use of the PanTum Detect blood test allows for the pre-selection of individuals with a high tumor probability (PPV = 82.12%) and offers the possibility of using imaging procedures to screen these preselected asymptomatic individuals for possible tumors - also under suitable cost and radiation aspects (10). Since TKTL1 expression is linked to increased glucose uptake into the cell, an increased glucose uptake is a clear indication of transformation of a benign to a malignant tumor. PET/CT technology exploits the process of increased glucose uptake by using radiolabeled glucose to visualize increased glucose uptake in cells. The crucial conversion of a benign to a malignant tumor is thus detectable using the PanTum Detect blood® test by measuring elevated levels of TKTL1 and can be confirmed by PET/CT through increased glucose uptake in morphologically conspicuous tissues. For this reason, the combination of a blood test and imaging techniques can be used to precisely detect premalignant lesions that are on the verge of becoming dangerous in their development.

Like any diagnostic tool, the PanTum Detect blood test also has some limitations which are outlined below. For example, a manifest cancer diagnosis or suspected cancer as well as treatment with immunosuppressants (e.g., corticosteroids), amygdalin intake, acute febrile diseases, active herpes zoster, vaccinations, intake of contrast agents, surgeries or serious injuries may influence the test results and thus represent temporary exclusion criteria.

At present, the test is solely applied in early cancer screening. It is currently not used in monitoring, staging or follow-up. In case of elevated values, imaging techniques such as MRI or PET/CT must be performed for verification and localization. In addition, multiple small individual events could potentially add up to an elevated blood test level, although these are not yet detectable by current imaging techniques due to insufficient size. However, in case these small lesions continue to grow and the test is performed regularly, they will be detectable on imaging in a timely manner.

In clinical practice, the Pantum Detect blood test offers the advantage of non-invasive *in vitro* diagnostics without significant side effects or risks. Blood collection can be easily performed by a physician and subsequent analysis by trained laboratory personnel. Moreover, this blood test offers the advantage of exploiting the specific elimination of tumor cells by the innate immune system without dilution effects, as antigens are detected directly in macrophages. In addition, the two biomarkers Apo10 and TKTL1 allow the detection of many different tumor entities - including those for which tumor markers are not yet available (10).

The case report presented here conclusively shows that the PanTum Detect blood test in combination with subsequent radiological imaging offers the possibility to detect tumors at an early, localized stage, when prospects of cure are still favorable. In the presented case, the blood test indicated a high tumor probability in an asymptomatic individual, with subsequent radiological imaging confirming this suspicion and detecting early-stage lung cancer. Further customization and expansion of current screening programs to include such blood tests and imaging techniques should be considered in the future.

## 4 Patient perspective

The patient describes the entire process - from the Pantum Detect blood test to PET/CT imaging to surgical resection - as “life-saving.” At the time of the blood test, she had no complaints and felt healthy. She positively emphasized that after the abnormal blood test, all further measures were taken quickly and thus the period of uncertainty was short. She is particularly pleased that, thanks to the blood test and subsequent imaging, the tumor was detected at such an early stage and completely removed that no chemotherapy was required, and she is considered cured. The Patient approved the publication of this case report.

## Data availability statement

The raw data supporting the conclusions of this article will be made available by the authors, without undue reservation.

## Ethics statement

The studies involving human participants were reviewed and approved by Ethik Kommission der Ärztekammer Hamburg Weidestrasse 122b 22083 Hamburg Germany. The patients/participants provided their written informed consent to participate in this study.

Written informed consent was obtained from the individual(s) for the publication of any potentially identifiable images or data included in this article.

## Author contributions

All authors contributed to the article and approved the submitted version. KF supervised the subject and performed the MRI examination. SB and AG drafted the final manuscript. RS performed the critical revision. RS and MG gave the final approval of the version to be published.
